# Cognitive and Relational Processes Associated to Mental Health in Italian High School Students during COVID-19 and Russian–Ukrainian War Outbreaks

**DOI:** 10.3390/ijerph21040508

**Published:** 2024-04-19

**Authors:** Attà Negri, Arianna Barazzetti, Alice Rinzivillo, Rachele Mariani, Cinzia Di Monte

**Affiliations:** 1 Department of Human and Social Sciences, University of Bergamo, 24129 Bergamo, Italy; arianna.barazzetti@unibg.it; 2 Independent Researcher, 65121 Pescara, Italy; alicerinz@live.it; 3 Department of Dynamic and Clinical Psychology and Health Studies “Sapienza”, University of Rome, 00185 Rome, Italy; rachele.mariani@uniroma1.it (R.M.); cinziadm92@hotmail.it (C.D.M.)

**Keywords:** COVID-19, Russian-Ukrainian war, pandemic resilience, core belief violation, meaning-making

## Abstract

The negative impact of the COVID-19 pandemic on mental health has been widely demonstrated; however, few studies have investigated the psychological processes involved in this impact, including core beliefs violation, meaning-making disruption, interpersonal support, or one’s relational functioning. This study explored the mental health of 215 Italian adolescents during the COVID-19 pandemic and the subsequent outbreak of the Russian–Ukrainian war. By administering a set of questionnaires, several cognitive and emotional variables were investigated, including core belief violation, meaning attribution to the pandemic and war, attachment, and emotion regulation, social media addiction, and relationships with significant others and teachers. We conducted some descriptive, mean difference, correlational, and predictive analyses that revealed a significant association between core belief violation caused by war and pandemic, ability to integrate war and pandemic within personal meaning universe, the relational support received, and mental health. The relationship with teachers during these challenging periods improved significantly according to the respondents’ opinion, becoming both more authoritative and empathic. This study offers insights into what cognitive and relational processes are useful to intervene on to reduce the distress of adolescents who are facing significant moments of crisis due to events that challenge their cognitive and emotional balance.

## 1. Introduction

In the recent past, there have been at least two significant crises in rapid succession worldwide. In 2020, the COVID-19 pandemic brought significant disruptions to our daily lives, leading to profound changes in education and social interactions worldwide. In general, widespread lockdowns, job losses, income reductions, and mobility restrictions were among the many challenges faced [1]. Amidst global recovery efforts, the Russian–Ukrainian war, which commenced on 24 February 2022, cast a shadow over post-pandemic economic prospects and triggered a humanitarian crisis across Europe [2,3]. Despite not being directly engaged in the ongoing war, Italy and its citizens are not exempt from the repercussions it entails. There is a palpable concern among Italian citizens, as well as those in other European nations, regarding the potential spread of the conflict beyond its current borders. Furthermore, the impact of the war can be observed through rising energy and commodity prices, as well as distressing scenes conveyed via the media, which predominantly focus on the ongoing conflict [4,5,6].

The war, alongside the COVID-19 pandemic, carries significant implications, as the experience and expression of negative emotions have the potential to impact the mental well-being of the population [7,8,9]. The detrimental impacts of war and terrorism on mental health have been examined in the past. Studies conducted in nations that have endured war and/or armed conflict have consistently demonstrated a marked decline in the mental well-being of populations directly affected by these events [10,11,12,13,14,15]. In addition, extensive media coverage has made the psychological repercussions of the conflict accessible worldwide, causing anxiety disorders, acute stress reactions, depressive episodes, and PTSD in various populations in the post-pandemic COVID-19 landscape, which had already generated numerous negative implications for individuals’ mental well-being [16,17,18,19,20].

Students are one of the demographic groups most affected by the COVID-19 pandemic outbreak and the Russian–Ukrainian war [2,3,21,22]. During the COVID-19 pandemic, schools underwent significant changes, shifting from traditional in-person classes to remote learning models due to lockdowns and social distancing measures. This transition to virtual classrooms presented challenges for both educators and students, necessitating adjustments in teaching methods and curriculum delivery [23]. The closure of schools also led to the loss of in-person interactions and extracurricular activities, impacting students’ social development and well-being [24,25,26,27,28,29,30]. Studies on the mental health of students consistently highlight the need for self-regulation and motivation in online learning environments [31,32,33,34]. Additionally, there has been an increase in mental health disorders such as Major Depressive Disorder and Generalized Anxiety Disorder among students, attributed to the pandemic’s effects on social distancing and uncertainties in educational procedures [35,36]. Prolonged exposure to a sense of helplessness, also known as learned helplessness, poses a risk factor for depression, especially considering the lingering psychological aftermath of COVID-19 [37,38]. Some authors maintained that the combined impact of COVID-19 and the Ukrainian war would pose a significant risk to the mental health of specific categories, such as women, adolescents, the older people, individuals with disabilities, and healthcare professionals [39]. The findings revealed the presence of negative emotions, such as anxiety, anger, and disgust, among the Italian population even if their involvement in the conflict was only indirect. These results have significant implications, as the experience and expression of negative emotions have the potential to impact the mental well-being of the population. Recent systematic literature reviews underlined the increase of symptoms related to anxiety, panic, depression, eating disorders, sleep disorders, social withdrawal, stress disorders, psychotic symptoms, anti-conservative thoughts, and self-harming acts also in adolescents, aggravated by COVID-19 restrictions and the impacts of Russian–Ukrainian war [2,3,7,8,38,40].

While traumatic or negative events have been linked to a range of negative physical and psychological outcomes, extensive research has also focused on the significance of personal and social resources, commonly referred to as protective factors, that can positively influence responses to such events. Researchers have identified various risk and protective factors that can affect mental health outcomes in the face of traumatic or negative events. Among the recognized protective factors, there are social support and the ability to make meaning of negative events which had challenged one’s core beliefs [41,42,43,44,45]. Protective factors can be both internal or external to the individual (e.g., family, qualified teachers, peer relations, and the community or individual social environment) [46,47]. Through social support, significant people can influence the individual’s capacity to deal with stressful experiences, cope well with these experiences, and positively face these challenges. Adolescent students especially need good social support to increase resilience when facing pressure or stress [48], and the presence of a supportive atmosphere can offer reassurance and foster a sense of security among students [49]. The optimal balance of distress varies and depends on individual predisposing factors. Few studies have proposed general explanation models that include the psychological variables responsible for the impact of direct and indirect stressors on mental health during the pandemic. For example, Milman and colleagues [18] developed an explanatory model to understand the relationship between pandemic-related stressors and their effects on mental health. The model is based on two primary psychological processes mediating these effects, namely, violation of core beliefs and disrupted meaning-making. Core beliefs are the fundamental beliefs individuals hold about the world, themselves, and their relationships, and stressful or traumatic events can violate these beliefs, causing disorientation and mental health disorders. Disrupted meaning-making occurs when individuals struggle to make sense of challenging events and integrate them into their worldview. Some studies [41,42,43,44,45] were conducted on the role of core belief violation and disrupted meaning-making in mental health during the pandemic. They found that these processes mediated the relationship between direct and indirect pandemic stressors and people’s depression severity, general anxiety, and coronavirus anxiety more than demographic factors and pandemic stressors combined. Compliance with social isolation measures was found to reduce the burden of the pandemic by reducing the impact on core beliefs associated with predictability and control. Overall, research has also shown that confinement at home and strengthened relationships can reduce core belief violation and facilitate functional meaning-making in well-functioning families. Negri et al. [42] confirmed Milman et al.’s [18] findings, showing that core belief violation, reduced meaning-making, and increased perception of vulnerability and mortality significantly influenced mental health symptoms during the pandemic. These factors mediated the relationship between COVID-19 stressors and mental health outcomes, indicating that individuals experiencing greater core belief violation and reduced meaning-making suffered more severe mental health issues.

Based on these considerations and on these studies that not only snapshot the consequences of the pandemic on people’s health but also explain the mechanisms that activate its negative impact, this study aimed to (a) detect whether these negative effects of the pandemic persist over time in the student population and are increased or not with the outbreak of the Russia–Ukraine war, (b) detect whether the possible negative effect of the war is also similarly associated with the cognitive factors of disruption of meaning-making and violation of core beliefs, (c) investigate whether other psychological factors more related to relationship functioning such as attachment and perceived support from significant others and teachers are also positively or negatively associated with students’ mental health, and (d) explore some predictive models of such impact including the cognitive and relational processes found to be significantly associated with it.

## 2. Materials and Methods

### 2.1. Participants and Procedure

Two hundreds and fifteen Italian high school students (46 of whom were male), ranging in age from 14 to 18 (M = 16.23; SD = 1.56), participated in the study. The participants attended two high schools in Italy, one in the north (*n* = 149) and one in the center (*n* = 66) of the country. Sociodemographic information can be found in Table 1.

Between May and June 2022, participants were recruited, and they completed questionnaires through an online form. Before they filled it out, the parents of participants received detailed information about the study. Only students whose parents have given their consent to participate received the online form to be completed with several questionnaires. Participation was voluntary, and confidentiality and anonymity were assured. The participants could withdraw from the study at any time. The study was approved by the Ethics Committee of Bergamo University (Minutes n. 04/2022 of the ethic committee held on 27 July 2022). The research was conducted according to the ethical guidelines for psychological research established by the Italian Psychological Association.

### 2.2. Measures

Participants were asked to complete online some questionnaires and questions to collect information about COVID-19-related stressors, psychological well-being and mental health, personal and sociodemographic information, use of social networks, attachment style, relationships with parents and teachers, core beliefs violation caused by war and pandemic outbreaks, and capacity for meaning-making. The instruments applied in the study are described in more detail below.

#### 2.2.1. Survey about COVID-19-Related Stressors and Personal Information

At the beginning of the administration, respondents answered questions about age, gender, educational institution, and year of secondary school attended. Students were also asked to say whether they had received a psychological diagnosis before the COVID-19 pandemic and whether they had received psychological support during the pandemic. Other questions included whether they had tested positive for COVID-19, whether they were vaccinated, whether they had an acquaintance or family member who had died from COVID-19, and months of distance attendance of school classes. The last two items were open-ended questions to be answered: “In general, what has the pandemic changed in the way you think, feel and live your life?” and “In general, how have the events of the war in Ukraine changed the way you think, feel and live your life?”

#### 2.2.2. Questionnaires on Psychological Factors

- *Core Belief Inventory* (CBI) [50]. It is a 9-item questionnaire that assesses if a person’s fundamental beliefs about the nature of the universe (as fair and controllable), the predictability of the future, a sense of purpose in life, one’s self-worth and identity, as well as spirituality and religion, have been violated by a certain event. On a 6-point scale ranging from “not at all” (0) to “to a very great degree” (5), participants reported how much an “event” led them to critically consider their essential beliefs. In this study, we tested how much two specific events, “the coronavirus pandemic” (CBI_COVID) and “Ukrainian war” (CBI_War), have questioned the core beliefs of respondents. Higher values suggest a more severe violation of core beliefs. In this study, CBI showed good internal consistency (α = 0.85 for COVID and 0.86 for War).
- *Integration of Stressful Life Experiences Scale—Short Form* (ISLES-SF) [51]. It is a brief scale (six items) that is an assessment of meaning made of stressful events, in this study, the pandemic (ISLES_COVID) and the Ukrainian war (ISLES_War). In this study, participants rated their level of agreement with the items using a five-point scale that ranges from “strongly disagree” (1) to “strongly agree” (5). A higher score indicates more disruption in meaning-making and the final score is calculated as the sum of the item scores. In this study, ISLES-SF showed acceptable internal consistency (α = 0.79 for COVID and 0.77 for War).
- *Relationship Questionnaire* (RQ) [52]. The RQ presents four brief descriptions of the main attachment styles, and the respondent is asked to say which of the four attachment models is most appropriate for describing their own relationships. In addition, for each model, the respondent is asked to give a score on a 7-point scale from “not at all like me” to “very much like me”. Two synthetic factors can be computed: the model of the self or attachment anxiety, and the model of other or attachment avoidance.
- *Emotion Regulation Questionnaire* (ERQ) [53,54]. It is a 10-item scale designed to measure respondents’ tendency to regulate their emotions in two ways: (1) Cognitive Reappraisal and (2) Expressive Suppression. Cognitive Reappraisal is defined as changing the way one thinks about a situation in order to change its emotional impact, and Expressive Suppression is conceptualized as inhibiting behavioral expressions of an emotion [34]. Respondents answered each item on a 7-point Likert-type scale ranging from 1 (strongly disagree) to 7 (strongly agree). In this study, ERQ showed acceptable internal consistency (α = 0.84 for Cognitive Reappraisal scale and α = 0.78 for Expressive Suppression scale).
- *The Network of Relationships Inventory: Behavioral Systems Version* (NRI-BSV) [55]. It is a questionnaire composed by 34 items about eight features of close relationships. The first five features (seeking in the other a safe refuge, seeking in the other a safe base, offering the other a safe base, friendship) make up the support factor, while the last three (conflict, antagonism, and criticism) make up the negative interaction factor. The instrument can be used to investigate the characteristics of relationships with different people. Specifically, the relationships considered are mother, father, same-sex friend, opposite-sex friend, romantic relationship, and significant other. The response mode consists of a 5-point Likert scale, from never to very much. In this study, the internal consistency values for the factors Support and Negative Interactions of NRI-BSV were good for all relationships (Cronbach’s mean α = 0.83; range 0.80–0.85).
- *Survey on perceived qualities of the relationship with schoolteachers*. We asked participants to talk about their relationship with teachers before the pandemic, during the pandemic, and in the last month, through six questions on some relevant qualities of the relationship with teachers. We, precisely, asked for a score about how much they did feel their teachers were authoritative, empathic, tolerant, allied, stimulating, and supportive. Each question is scored on a 5-point Likert scale that ranges from 0 (nothing) to 5 (very much). Two difference scores were calculated between the ratings given for the periods during and before the pandemic (Δ_a_) and between the rating for the last month period and before the pandemic (Δ_b_). When the difference scores were positive, there was an increase in scores; when they were negative, a decrease.
- *Vulnerability and mortality perception*. Two individual questions were aimed at asking respondents how much more vulnerable and fragile they felt because of the pandemic (Vulnerability COVID) and the war (Vulnerability War) and how much more the pandemic and the war made them think about their own death (Mortality COVID and Mortality War).

#### 2.2.3. Psychological Health

- *Self-reported risk behaviors survey*. We asked participants about their risk-taking behaviors before the pandemic, during the pandemic, and in the last month. We, precisely, asked about how many times they injured themselves, and how many times they used drugs or alcohol to the point of feeling ill. Each question is scored on a 5-point Likert scale that ranges from 0 (never) to 5 (more than 3 times). Two difference scores were calculated between the ratings given for the periods during and before the pandemic (Δ_1_) and between the rating for the last month period and before the pandemic (Δ_2_). When the difference scores were positive, there was an increase in scores; when they were negative, a decrease.
- *Satisfaction with Life Scale* (SWLS) [56]. It is the scale that is most frequently used to measure life satisfaction among different populations. A five-item Likert scale with 1 being strongly disagreed and 5 being strongly agreed is used to rate the five items. Item mean scores are calculated by dividing the total score by five. Low scores indicate a low life satisfaction, whereas high scores indicate a high degree of life satisfaction. In this study, SWLS showed good internal consistency (α = 0.85).
- *Bergen Social Media Addiction Scale* (BSMAS) [57]. This instrument was used to evaluate problematic social media use (e.g., “How often during the last year have you spent a lot of time thinking about social media or planned use of social media?”). It is a six-item scale that rates salience, mood modification, tolerance, withdrawal, conflict, and relapse on a 5-point Likert scale from 1 (very seldom) to 5 (very often). The higher the sum score, the higher the level of addictive social media use. In this study, BSMAS showed good internal consistency (α = 0.88).
- *Four-item Patient Health Questionnaire* (PHQ-4) [58]. The PHQ-4 is an ultra-brief tool for detecting both depression and anxiety symptoms experienced over the last two weeks. It has two 2-item subscales: one for anxiety and one for depression. Each item is scored on a 4-point Likert scale that ranges from 0 (not at all) to 3 (nearly every day). The range of the PHQ-4’s overall score is 0 to 12. Higher scores indicate higher levels of anxiety and depression. In this study, PHQ showed suitable internal consistency (α = 0.80).
- *General Population—Clinical Outcomes in Routine Evaluation* (GP-CORE) [59]. It is a 14-item scale that was developed from the Clinical Outcomes in Routine Evaluation-Outcome Measure (CORE-OM) to measure psychological distress in a non-clinical population. The items concern well-being, problems, and psychological functioning. On a Likert-type scale, respondents rate how frequently they felt a particular way in the course of the week before, ranging from 0 (not at all) to 4 (most or all of the time). Greater well-being is indicated by lower scores. In this study, GP-CORE showed good internal consistency (α = 0.84).

### 2.3. Statistical Analyses Overview

To explore the associations between respondents’ mental health indices and cognitive, emotional, relational, and COVID-19-related variables, we conducted four different types of analyses. First, through descriptive analyses of the data collected in each measured variable, we outlined a picture of the mental health of the participating students and of the main variables associated with it. Second, a set of analyses of variance (ANOVAs) was conducted to test the presence of significant differences in the means of subgroups of participants defined by the dichotomous variables (gender, COVID-19 deaths, psychological support during the pandemic, COVID-19 diagnosis) and the average values at different times in risk behaviors and perceived relationship qualities scores. Third, by correlational analysis, we tested the association between the cognitive and relational variables and the mental health indices. Fourth, changes over time (pre-COVID, during COVID, and in recent months) in self-reported risk behavior and relationship with teacher scores were tested with a series of repeated-measures ANOVAs. Fifth, for each mental health index, we performed a multiple regression analysis to find among the variables correlated to each index which could be considered also predictors of mental health levels. We conducted both correlational analysis and multiple regression analyses, including in the models the general satisfaction with life (SWLS) as a covariate to keep controlled the effect of this variable. Effect sizes (η^2^, eta-squared) were calculated to determine the magnitude of differences between groups in the ANOVA analyses.

## 3. Results

### 3.1. Participants’ Descriptive Features

As shown in Table 1, most of the respondents were female (78.6 percent) and of Italian nationality (99.6 percent). They were evenly distributed among the five secondary school years, attended in Italy by students between the ages of 14 and 18. The average age of the respondents was 16.23 years (SD = 1.56).

Regarding factors related to COVID-19, participants attended school classes remotely for 9.2 months on average, with significant variability (SD = 4.06). Most of the students (55.8%) either did not test positive for COVID-19 or tested positive but were asymptomatic (13.0%); 67 out of 215 students (31.2%) tested positive and presented symptoms produced by the virus. A not insignificant proportion of students experienced the loss of a family member due to COVID (13%) or an acquaintance (44.2%), while 42.8% of respondents did not have a COVID-related death.

Variables measuring mental health showed an important level of psychological suffering at questionnaire administration (May–June 2022): 6.3% of students underwent psychological treatment during the pandemic and 4.2% took psychotropic drugs; respondents’ average score on GP-CORE was equal to 1.66, above the clinical cut-off (Female = 1.63; Male = 1.49)*;* the PHQ-4 mean (5.18) was between mild and moderate levels (normal = 0–2, mild = 3–5, moderate = 6–8, severe = 9–12); the average score on BSMAS was equal to 16.37, below the clinical cut-off of 24; and finally, the students reported that in the last months before the administration of the questionnaires they had “rarely” or “sometimes” put themselves in dangerous situations, or they had harmed themselves or they had taken drugs or alcohol to the point of feeling ill (M = 1.40; range: form 0 = never to 3 = often). In terms of life satisfaction, respondents achieved a mean score of 22.11 with a standard deviation of 6.61; in general, they were slightly satisfied, but the variability of the scores also covers some dissatisfaction (extremely satisfied = 31–35; satisfied = 26–30; slightly satisfied = 21–25; neutral = 20; slightly dissatisfied = 15–19; dissatisfied = 10–14; extremely dissatisfied = 5–9).

Regarding psychological factors that may explain the negative impact on mental health of the stressful events of the pandemic and the war between Russia and Ukraine, the responses indicate that both events challenged the respondents’ beliefs (CBI), but the pandemic (M = 2.52 above the mean response scale score of 2.50) did so more than the war (M = 1.85). The ability to make sense of these two events (ISLES) was also difficult for the respondents (M = 15.56 for the pandemic and M = 15.95 for the war, just above the mean score of the response scale), and indeed in this case the outbreak of the war between Russia and Ukraine was an event slightly less able to be integrated into one’s horizon of meaning. Furthermore, only 19.9 percent of the students recognized themselves in a secure attachment style, while among the others the most widespread attachment style was the fearful one. The emotion regulation strategies of Cognitive Reappraisal and Expressive Suppression, as measured by the Emotion Regulation Questionnaire, are partially used by the respondents, the latter being the most frequently used (respectively M = 22.14 on 6 items and M = 15.07 and 4 item with range 1–7).

### 3.2. The Possible Role Played by Gender, Psychological Support, and COVID-19-Related Variables

Male and female students showed different scores in many variables (See Table 2). The magnitude of the differences (η^2^) could be considered high or medium in most of the comparisons according to Cohen’s [60] interpretive guidance.

From a mental health perspective, females have significantly lower levels of general life satisfaction than males and had significantly higher levels of general malaise (GP-CORE), anxiety and depression (PHQ-4), and social media use addiction (BSMAS).

It is again female students who, in comparison with male students, felt their vulnerability and mortality more because of the events of the pandemic and the Russia–Ukraine war (Vulnerability and Mortality) and showed more difficulty in integrating these events into their personal value system (ISLES), because they challenged their core beliefs more than males (CBI).

On the relational side, females gave higher scores in attachment avoidance and anxiety; particularly, they recognized themselves more in the fearful attachment pattern. However, female students felt more relational support from their parents and same-sex friends.

Another variable that significantly differentiates participants’ scores is whether they received psychological support from a professional during the pandemic (See Table 3). The magnitude of the differences (η^2^) could be considered medium or small in most of the comparisons according to Cohen’s [61] interpretive guidance.

On average, those who underwent a psychological treatment did not show a more pronounced general discomfort profile (GP-CORE and PHQ-4) than other students but only a lower life dissatisfaction score (SWLS) and higher dysfunctional use of social media. However, in terms of self-reported risk behaviors, those who underwent psychological treatment significantly gave much higher scores than those who did not feel the need for professional psychological support. Moreover, on average, those who received psychological support had attended school classes for more months remotely.

The attachment of the group of those who received psychological support tended to be more problematic in terms of anxiety, and the fearful attachment pattern was more frequent than the other students. On the positive side, these students saw a more pronounced improvement in perceived qualities of the relationship with their teachers (Δ_b_).

Those who obtained help from a psychological professional during the pandemic had, on average, more pronounced perceptions of vulnerability and mortality in the face of the war and pandemic events, and these events challenged these students’ core beliefs more.

Respondents who tested positive for COVID-19, regardless of whether they presented the corresponding symptoms, had significantly worse mental health scores (GP-CORE, PHQ4, and BSMAS) than students who did not test positive (see Table 4). The values of effect size (η^2^) were small, according to Cohen’s [61] interpretive guidance.

Finally, having had a death among one’s family members or acquaintances compared to those who did not also led to significant differences in respondents’ scores (See Table 5). Also in this case, the magnitude of the differences was small in all comparisons, according to Cohen’s [60] interpretive guidance.

Those who experienced losses struggled significantly more to integrate the war and the pandemic into their horizon of meanings (ISLES), thought more about their own fragility and mortality, and saw their core beliefs challenged more (CBI).

Those who experienced losses due to COVID-19 reported both higher scores in self-reported risk behaviors and a lower pre-post-pandemic delta in these behaviors (Δ_2_) than those who did not experience bereavements, i.e., the scores of these students decreased less than those who did not lose any loved ones or acquaintances due to COVID-19.

Lastly, there were no differences in any variables between those who received the vaccine and those who did not, between those who had a psychiatric diagnosis before the pandemic and those who did not, and between those who took psychotropic drugs during the pandemic period and those who did not.

### 3.3. The Possible Role Played by Months of Remote Schooling and Cognitive and Relational Processes

To test their possible role played in students’ mental health, we calculated partial correlation coefficients between months of remote school attendance and cognitive and relational processes that we believe are explanatory in the negative impact on well-being on the one hand and mental health indices on the other. We calculated the coefficients by eliminating any association with level of satisfaction with life (SWLS) that may affect the variables included in the analysis.

The length of the remote schooling was positively correlated with self-reported behavior in the last month but not with the other indexes of mental health (Table 6).

A clear and recurring association emerged: the higher the scores on violation of core beliefs (CBI), disruption of meaning-making ability (ISLES), and feelings of mortality and frailty, the higher the scores on depression and anxiety (PHQ-4), social media addiction (BSMAS), and general malaise (GP-CORE), as shown in Table 6. This association was observed for both the pandemic and the war event.

As for emotion regulation (ERQ), there was a characteristic trend: the more the Cognitive Reappraisal strategy was present, the less there was dysfunctional use of social media (BSMAS), while the greater the presence of Expressive Suppression, the worse were the indices of depression and anxiety (PHQ-4) and general malaise (GP-CORE) (Table 6).

The Cognitive Reappraisal strategy was also associated with greater scores of self-reported risk behaviors during the pandemic and in the last month. Finally, the violation of the core beliefs due to the outbreak of the pandemic and the inability to make sense of this event in one’s value horizon, together with the more vivid thought of one’s own mortality, were associated with more self-reported risk behaviors during the pandemic period (Table 6).

Looking at relational factors, we see that the greater the attachment anxiety, the worse the mental health scores (PHQ-4 and GP-CORE) (Table 7). In particular, it was the preoccupied and fearful attachment styles that were associated with higher levels of anxiety, depression, and general malaise, and in the case of fearful attachment, also with dysfunctional use of social media (BSMAS). Attachment characteristics, on the other hand, appear not to be associated with risk behaviors. The dismissive attachment style did not correlate with both mental health indices and risk behaviors.

Regarding perceptions about relationships, the most important role was played by parents, friends, and partners, although in different ways (Table 7). In general, the levels of general malaise (GP-CORE), depression, anxiety (PHQ-4), social media addiction (BSMAS), and self-reported risk behaviors increased in correspondence with greater negative interactions with both the mother and father.

If we look at the relationships with same-sex friend or opposite-sex friend or even with romantic partner, the worst mental health scores and risk behaviors increased along with both perceived support from and negative interactions with them. In contrast, the quality of the relationship with teachers showed no significant associations with their students’ mental health, at least in their opinion (Table 7).

### 3.4. Changes in Risk Behaviors and Relationships with Schoolteachers

The comparison between the mean scores of the items on self-reported risk behavior in the pre-COVID period (M = 1.23, SD = 1.80), during the COVID period (M = 1.33, SD = 2.29), and in the last few months (M = 1.39, SD = 2.44) showed no significant differences. The relationship with teachers, on the other hand, underwent significant changes as reported by the respondents: the positive qualities of the relationship increased significantly from pre-COVID (M = 15.82, SD = 5.86), COVID (M = 17.18, SD = 5.78), and in the last month (M = 18.46, SD = 4.46) (pre-COVID vs. during COVID: t = −5.056, df = 214, *p* ≤ 0.001; during COVID vs. last month: t = −4.226, df = 214, *p* ≤ 0.00). Of all the variables measured, only the anxious style is predictive of a positive increase in teacher relationship quality from pre-pandemic to last month (β = 0.168, t = 2.488, *p* = 0.004, R^2^ 0.028).

### 3.5. The Predictors of Mental Health

Five multiple linear regression models were performed to investigate possible predictors of student’s mental health as measured by GP-CORE, PHQ-4, BSMAS, and the self-reported risk behaviors index during the pandemic and in the last month (Table 8).

The most complete model we found was the one with the level of general malaise as measured by GP-CORE as the dependent variable. The predictors found to be significant in order of importance were (a) feeling vulnerable due to the pandemic, (b) difficulty in making sense of the pandemic (ISLES), (c) use of the strategy of Expressive Suppression of emotions (ERQ), (d) being positive on the test for COVID-19, (e) negative interactions with the father (NRI-BSV), (f) attachment avoidance (RQ), (g) lower emotional support from the father and (h) from the romantic partner (NRI-BSV). This model predicted 56.6% of GP-CORE scores (R^2^ = 0.566; adjusted R^2^ = 0.550; F = 33.640 *p* < 0.001).

A second linear regression analysis had the level of depression and anxiety as measured by PHQ-4 as the dependent variable and (a) perception of vulnerability due to pandemic, (b) perceived negative interaction with father (NRI-BSV), (c) difficulty in meaning-making about war (ISLES), (d) violation of core beliefs for pandemic (CBI), and (e) months of distance learning as significant predictors. This model predicted 43.5% of PHQ-4 scores (R^2^ = 0.435; adjusted R^2^ = 0.418; F = 26.638, *p* < 0.001).

A third model tested the significant predictors of risk behaviors during the pandemic. Only a combination of negative interactions with partner and father (NRI-BSV) and core beliefs violation for pandemic was found to predict risk behaviors during pandemic. This model predicted 14,8% of risk behaviors during pandemic (R^2^ = 0.148; adjusted R^2^ = 0.136; F = 12.261, *p* < 0.001).

A last model of linear regression with self-reported risk behaviors in the last month as a dependent variable was tested. In this model, only the negative interactions with partner and father (NRI-BSV) resulted as significant predictors. This model was significant and predicted 11.5% (R^2^ = 0.115; adjusted R^2^ = 0.106; F =13.712, *p* < 0.001).

Social media addiction scores (BSMAS) were not predicted by any of the variables considered in the present study.

## 4. Discussion

This study drew a less than optimistic picture of the mental health of Italian students during the period marked by two consecutive crises, the outbreak of the pandemic and the outbreak of the Russia–Ukraine war. The mean scores revealed the presence of mild to moderate levels of depression and anxiety (PHQ-4), a level of malaise above the clinical cut-off (GP-CORE), the presence at least sometimes of risk behaviors such as drug abuse, alcohol, or self-injurious behaviors, and satisfaction with one’s life only slightly toward the positive side. This is even though only 5.1% of respondents had received a psychiatric diagnosis even before the pandemic and only 4.2% took psychotropic drugs during the pandemic period. In addition, 16.3% felt the need to seek psychological support from a professional during the pandemic period. Thus, regarding the first (a) aim we had set in the present study, we can say that the psychological malaise that began with the pandemic persisted and was maintained even during the initial period of the outbreak of war in Ukraine.

These are not surprising data, since direct and indirect stressors related to the COVID-19 pandemic were important: 44.2% of students tested positive for COVID-19 and 31.2% also had symptoms related to the virus, in line with epidemiological data [62,63]; most students (57.2%) had a relative or acquaintance who had died due to COVID-19, a figure that unfortunately matched the general statistics [63]; and finally, participants attended school classes remotely for 9.2 months on average, i.e., about one year of school. The combination of these stressors has certainly had an important negative impact on students’ health as much literature has well documented e.g., [60,64].

As a part of the literature highlighted [18,41,42,43,44,45], also in this study we found a close association between worse scores on mental health dimensions and cognitive factors such as violation of core beliefs (CBI), disruption of meaning-making ability (ISLES), and a heightened sensation of vulnerability and mortality. This suggests an explanatory role of these psychological factors in the relationship between stressors and psychological distress. In other words, a stressful event such as a pandemic or war has a negative impact to the extent that personal meaning attribution processes are challenged. As hypothesized (see aim (b) of this study), this seems to apply not only to the outbreak of the pandemic, which was a very impactful event on people’s lives, but also to the outbreak of the Russia–Ukraine war, at least in Italy, where people had been used to having for many years a peaceful relationship between the major geopolitical blocs in the Eurasian area, as some recent studies demonstrated [2,3,4,7,8,10,39,40]. In fact, our data say that war made it difficult for students to integrate and make sense of the war event in their cognitive universe of life, similarly to pandemic (ISLES: M = 15.56 and 15.95, respectively, for pandemic and war); the same happened for the violation of basic beliefs although to a lesser extent than pandemic, which involved people more directly and personally (CBI: M = 2.52 and 1.85 respectively for pandemic and war). The war, thus, seems to have had a similar or even inferior effect to that of the pandemic, despite being an event that puts one’s own life and that of one’s family members at less direct risk. In addition, correlations were very high, consistent, and positive between all indices of mental health (PHQ-4, BSMAS, GP-CORE, risky behaviors) and core belief violation (CBI), difficulty in meaning-making capacity (ISLES), and feelings of mortality and vulnerability. Interesting, too, from a clinical perspective is that the Expressive Suppression strategy (ERQ) is associated more with increased general malaise (GP-CORE) and levels of depression and anxiety (PHQ-4), while the Cognitive Reappraisal strategy (ERQ) is associated more with social media addiction (BSMAS) and risky behaviors. Thus, a more internalizing or more externalizing profile of distress seems to emerge.

A third aim (see aim (c)) of the present study was to explore the role of relational factors in addition to cognitive factors in the impact of negative events on mental health. In this regard, it appears to be noteworthy the association between relationship factors and students’ mental health. In our opinion, this is the original aspect of present study, which sought to add to the cognitive psychological aspects already investigated in the literature by involving relational functioning through a questionnaire on attachment style (RQ), a very complete though little-used one on perceptions regarding relationships with significant others (NRI-BSV), and finally some questions on perceptions of relationship qualities with teachers. Surprisingly, only 19.9% of the students recognized themselves in a secure attachment style, while the most widespread attachment style was the fearful one. We do not know how accurate the respondents’ perceptions are, and thus we cannot say whether 80% of the students actually have a disturbed attachment style or whether it is the stressful situations of the pandemic and war that stimulated the activation of anxious and avoidant elements in the respondents. Certainly, the search for attachment becomes more pronounced at these times, and it is not always possible to find partners with whom to establish secure bonds. Schoolteachers in our study generally represented reference points for our students, and the relationship with them during the pandemic improved by becoming more authoritative, empathic, tolerant, allied, stimulating, and supportive, especially for students who identified themselves with a fearful attachment style. Positive relationships with teachers, however, did not correlate with mental health indices, either positively or negatively. This may be explained by the fact that teachers are surely a help and support for students’ growth, but they do not become as emotionally important people as family members do. In fact, correlation analysis showed that a very important role in maintaining good psychological balance during the pandemic and outbreak period was played by the support received from parents, a romantic partner, and friends (NRI-BSV); in contrast, negative interactions with them led to worse levels of mental health both in terms of general malaise, anxiety and depression, and risky behaviors. All these results point to the crucial role played by the relationships in construing, maintaining, and protecting a good mental health [48,49]. Surprisingly, the most pronounced role among these, either in terms of support or, at the opposite end, of negative interactions is played by the father. He is a less studied figure than, for example, the mother, but in systemic, direct, or indirect terms, he seems to be equally or more important than the other figures in the family as suggested but some scholars and research studies, e.g., [65,66].

Finally, as we proposed in the last (d) aim of this study, the results also offer predictive models of mental health in stressful situations such as those of pandemic and war, at least in the student population. Being female, testing positive for COVID-19, and having had a COVID-19-related death among acquaintances and family members were factors that led to worse levels of mental health. General malaise as measured by the GP-CORE was predicted by a combination of factors including feeling vulnerable due to the pandemic, experiencing difficulty in making sense of the pandemic (ISLES), using the strategy of Expressive Suppression of emotions (ERQ), being positive on the test for COVID-19, having negative interactions with the father (NRI-BSV), having attachment avoidance (RQ), and having lower emotional support from the father and romantic partner (NRI-BSV). High levels of anxiety and depression (PHQ-4) were predicted by similar factors: perception of vulnerability due to pandemic, perceived negative interaction with father (NRI-BSV), difficulty in meaning-making about war (ISLES), violation of core beliefs for pandemic (CBI), and months of distance learning. Risk behaviors, on the other hand, were predicted more by negative interactions with the partner and father (NRI-BSV). Finally, social media dependence scores (BSMAS) were not predicted by any of the variables considered in the present study. These predictive models give us a picture of the factors involved in students’ mental health in this time of pandemic and war. We find such a picture more inclusive—and therefore more useful for intervention—than the many models in the literature did, e.g., [67,68,69], both in terms of the variety of mental health outcomes considered and the cognitive and relational factors involved.

Some of the present study’s limitations in interpreting the results must be mentioned. This study utilizes a cross-sectional design, gathering data at a specific moment, which constrains the establishment of causation and tracking dynamic shifts over time. This study provides a snapshot of participants’ experiences and mental health status at a specific point in time, without the ability to track changes or assess long-term effects. Future research employing longitudinal designs is warranted to comprehensively explore the ongoing impacts of significant events like the War-on-COVID on mental health outcomes. Another constraint pertains to the sample’s representativeness, predominantly composed of high school students from specific Italian regions. This composition may curtail the applicability of findings to more extensive demographic cohorts or diverse geographic settings. Moreover, the sample enrolled in this study shows a good distribution for years of study, but there appears to be a greater representation of women. However, this finding is consistent with other studies in which female participation in research is consistently higher than males. The higher engagement of young women in psychological research has been well documented in the literature [70,71] and this implies a caution to generalize the results to an entire adolescent population. Finally, a last caveat in the interpretation of the results stems from the fact that we asked for ratings based on respondents’ memories of different past times. This surely may have led to bias due to memory issues or emotional and subjective contingencies of the respondents. Surely having more external and objective measures of adolescents’ mental health during the outbreak of the pandemic and war would have given us a more complete picture. However, the mere fact that respondents reported malaise at the time they were answering is important from a psychological perspective because subjective experience is the most relevant part that affects mental health, beyond the actual aspects of life. It would also be important to conduct studies that delve more deeply into how this impact takes shape in the particular stage of development that is adolescence.

## 5. Conclusions

In the wake of the unprecedented challenges presented by the COVID-19 pandemic and the subsequent Russian–Ukrainian war, this study sought to study the intricate network of psychological impacts on a cohort of Italian high school students. The findings underscore a complex interplay of factors influencing mental well-being, ranging from the direct consequences of the pandemic and war to individual coping mechanisms and social dynamics. The study reveals a substantial psychological toll inflicted by the dual crises, manifesting in high levels of malaise, depression, anxiety, and risky behaviors. Distinct correlations emerged, shedding light on the multifaceted nature of the challenges faced by the participants. Notably, the perceived violation of core beliefs during the pandemic and war and disruptions in meaning-making were identified as significant contributors to mental health outcomes. The study emphasizes that the optimal balance of distress varies based on individual predisposing factors, highlighting the importance of personalized approaches in mental health interventions. In the context of the ongoing global recovery efforts, the study calls for an understanding of the psychological repercussions of not only the pandemic but also concurrent geopolitical events. The integration of comprehensive models, such as the one proposed by Milman et al. [18] and extended by Negri et al. [42], provides a robust framework for comprehending the intricate relationships between cognitive, emotional, and social variables in shaping mental health outcomes.

This study contributes to the evolving discourse on mental health during complex and protracted crises, offering valuable insights for psychoeducational interventions in school contexts. Our findings offer insights into what cognitive and relational processes are useful to intervene in school settings and in general with people who are facing significant moments of crisis due to events that challenge their cognitive and emotional balance. It appears very important, for example, to foster students’ capacity in making a personal or collective sense to stressful and unexpected events such as the COVID-19 pandemic and war, to incorporate emotional intelligence instruction into school programs to improve students’ ability to comprehend and manage their emotions effectively, and to organize events or community forums that openly discuss the meaning through which to interpret events happening in society.

## Figures and Tables

**Table 1:** Descriptive statistics about the main sociodemographic, COVID-19-related, mental health, and psychological factor variables.

Sociodemographic Variables		Covid-Related Variables	
** Age**	**M (SD)**	** Months of remote schooling**	**M (SD)**
	16.23 (1.56)		9.2 (4.06)
** Gender**	***n*. (%)**	** COVID-19 positive test**	***n*. (%)**
Female	169 (78.6)	No	120 (55.8)
Male	46 (21.4)	Yes, asymptomatic	28 (13.0)
** Nationality**	***n*. (%)**	Yes, with symptoms	67 (31.2)
Italian	212 (98.6)	** COVID deaths**	***n*. (%)**
Non-Italian	3 (1.4)	No	92 (42.8)
** Year of High School**	***n*. (%)**	Death of an acquaintance	95 (44.2)
1st	61 (28.4)	Death of a family member	28 (13.0)
2nd	39 (18.1)	**Psychological Factors**	
3rd	35 (16.3)	** CBI**	**M (SD)**
4th	47 (21.9)	COVID	2.52 (1.18)
5th	33 (15.3)	War	1.85 (1.14)
**Mental Health Indices**	**M (SD)**	** ISLES**	**M (SD)**
** Diagnosis before pandemic**	***n*. (%)**	COVID	15.56 (5.43)
No psychiatric diagnosis	172 (80.0)	War	15.96 (5.13)
Specific learning disorders	32 (14.9)	** RQ**	***n*. (%)**
Other Psychiatric diagnoses	11 (5.1)	Secure attachment	43 (19.9)
**Psychological support during pandemic**		Fearful attachment	82 (38.0)
No	180 (83.7)	Preoccupied attachment	53 (24.5)
Yes	35 (16.3)	Dismissive attachment	37 (17.1)
** Psychotropic drugs during pandemic**			**M (SD)**
No	206 (95.8)	Secure attachment	3.04 (1.69)
Yes	9 (4.2)	Fearful attachment	3.99 (1.83)
** GP-CORE**	1.66 (0.73)	Preoccupied attachment	3.46 (1.85)
** PHQ-4**	5.18 (3.08)	Dismissive attachment	3.14 (1.77)
** SWLS**	22.11 (6.61)	RQ—attachment anxiety	1.27 (3.91)
** BSMAS**	16.37 (5.51)	RQ—attachment avoidance	0.63 (3.95)
** Self-reported risk behaviors**	**M (SD)**	** ERQ**	**M (SD)**
Before pandemic	1.23 (1.80)	Cognitive Reappraisal	22.14 (6.18)
During pandemic	1.33 (2.30)	Expressive Suppression	15.07 (5.31)
Last month	1.40 (2.45)		

Notes: SWLS = Satisfaction with Life Scale; GP-CORE = General Population–Clinical Outcomes in Routine Evaluation; PHQ-4 = Four-item Patient Health Questionnaire; BSMAS = Bergen Social Media Addiction Scale; CBI = Core Belief Inventory; ISLES = Integration of Stressful Life Experiences Scale; RQ = Relationship Questionnaire.

**Table 2:** Significant differences between Females (F) and Males (M).

	Mean	F	*p*	η^2^
CBI—COVID	M = 1.77; **F = 2.71**	25.016	<0.001	0.106
CBI—War	M = 1.45; **F = 1.95**	7.166	0.008	0.101
ISLES—COVID	M = 11.53; **F = 16.63**	36.336	<0.001	0.146
ISLES—War	M = 12.40; **F = 16.95**	32.367	<0.001	0.132
Vulnerability—COVID	M = 0.93; **F = 2.76**	39.837	<0.001	0.158
Vulnerability—War	M = 0.64; **F = 1.65**	15.364	<0.001	0.068
Mortality—COVID	M = 0.76; **F = 2.05**	20.411	<0.001	0.088
Mortality—War	M = 0.80; **F = 2.04**	18.201	<0.001	0.079
RQ—fearful syle	M = 3.00; **F = 4.25**	17.860	<0.001	0.078
RQ—attachment anxiety	M = 2.96; **F = 3.20**	6.203	0.014	0.028
RQ—attachment avoidance	M = −0.42; **F = 0.91**	4.061	0.045	0.003
NRI-BSV—Mother Support	M = 2.77; **F = 3.40**	18.028	<0.001	0.078
NRI-BSV—Father Support	M = 2.48; **F = 2.82**	4.360	0.038	0.020
NRI-BSV—Same-Sex Friend Support	M = 3.21; **F = 3.80**	15.306	<0.001	0.067
SWLS	**M = 24.53**; F = 21.56	7.609	0.006	0.035
GP-CORE	M = 1.26; **F = 1.76**	18.583	<0.001	0.081
PHQ-4	M = 3.27; **F = 5.66**	23.846	<0.001	0.101
BSMAS	M = 13.58; **F = 17.09**	15.379	<0.001	0.068

Notes: CBI = Core Belief Inventory; ISLES = Integration of Stressful Life Experiences Scale; RQ = Relationship Questionnaire; NRI-BSV = The Network of Relationships Inventory: Behavioral Systems Version; SWLS = Satisfaction with Life Scale; GP-CORE = General Population–Clinical Outcomes in Routine Evaluation; PHQ-4 = Four-item Patient Health Questionnaire; BSMAS = Bergen Social Media Addiction Scale. The higher mean value in the comparison is written in bold.

**Table 3:** Significant differences between those who received psychological support during the pandemic (Psy) and those who did not (noPsy).

	Mean	F	*p*	η^2^
Months of remote schooling	**Psy = 10.69**; noPsy = 8.92	5.666	0.018	0.26
CBI—COVID	**Psy = 16.29**; noPsy = 15.42	9.723	0.002	0.044
CBI—War	**Psy = 16.14**; noPsy = 15.92	9.091	0.003	0.041
Vulnerability—COVID	**Psy = 3.06**; noPsy = 2.23	5.739	0.017	0.026
Vulnerability—War	**Psy = 2.09**; noPsy = 1.31	7.349	0.007	0.033
Mortality—COVID	**Psy = 2.60**; noPsy = 1.63	8.861	0.003	0.040
Mortality—War	**Psy = 2.40**; noPsy = 1.66	5.109	0.025	0.023
RQ—fearful syle	**Psy = 4.57**; noPsy = 3.87	4.368	0.038	0.020
RQ—attachment anxiety	**Psy = 2.60**; noPsy = 1.01	4.930	0.027	0.023
Δ_b—_Relarionship with teachers	**Psy = 4.20**; noPsy = 2.34	4.327	0.039	0.020
Risky behaviors—pre-pandemic	**Psy = 2.31**; noPsy = 1.02	15.932	<0.001	0.070
Risky behaviors—during pandemic	**Psy = 3.11**; noPsy = 0.98	28.499	<0.001	0.118
Risky behaviors—last month	**Psy = 2.77**; noPsy = 1.12	14.049	<0.001	0.062
SWLS	Psy = 20.09; **noPsy = 22.50**	3.964	0.048	0.018
BSMAS	**Psy = 18.37**; noPsy = 15.98	5.622	0.019	0.026

Notes: CBI = Core Belief Inventory; RQ = Relationship Questionnaire; Δ_b_ = difference between scores given to last month and pre-pandemic period; SWLS = Satisfaction with Life Scale; BSMAS = Bergen Social Media Addiction Scale. The higher mean value in the comparison is written in bold.

**Table 4:** Significant differences between those who tested positive for COVID-19 (Cov) and those who did not (noCov).

	Mean	F	*p*	η^2^
GP-CORE	**Cov = 1.92**; noCov = 1.55	12.148	<0.001	0.054
PHQ-4	**Cov = 6.13**; noCov = 4.74	9.789	0.002	0.044
BSMAS	**Cov = 17.57**; noCov = 15.83	4.656	0.032	0.021

Notes: GP-CORE = General Population—Clinical Outcomes in Routine Evaluation; PHQ-4 = Four-item Patient Health Questionnaire; BSMAS = Bergen Social Media Addiction Scale. The higher mean value in the comparison is written in bold.

**Table 5:** Significant differences between those who have had an acquaintance or family member die due to COVID-19 (Deaths) and those who have not (NoDeaths).

	Mean	F	*p*	η^2^
CBI—COVID	**Deaths = 2.68**; NoDeaths = 2.28	6.081	0.014	0.028
CBI—War	**Deaths = 2.03**; NoDeaths = 1.60	7.591	0.006	0.034
ISLES—COVID	**Deaths = 16.54**; NoDeaths = 14.25	9.702	0.002	0.044
ISLES—War	**Deaths = 16.48**; NoDeaths = 15.26	3.003	0.085	0.014
Vulnerability—COVID	**Deaths = 2.61**; NoDeaths = 2.04	4.851	0.029	0.022
Mortality—COVID	**Deaths = 2.02**; NoDeaths = 1.49	4.643	0.032	0.021
Risk behaviors during pandemic	**Deaths = 1.61**; NoDeaths = 0.95	4.329	0.039	0.020
Δ_2—_Risk behaviors	Deaths = −0.16; **NoDeaths = 0.58**	7.101	0.008	0.048

Notes: CBI = Core Belief Inventory; ISLES = Integration of Stressful Life Experiences Scale; Δ_2_ = difference between scores given to the last month and the pre-pandemic period. The higher mean value in the comparison is written in bold.

**Table 6:** Partial correlation coefficients between mental health variables, months of remote schooling, and cognitive processes, controlled for satisfaction for life (SWLS).

	GP-CORE	PHQ-4	BSMAS	Risk Behaviors Pre-Pandemic	Risk Behaviors During Pandemic	Risk Behaviors Last Month
Months of remote schooling	0.039	0.099	0.097	0.004	0.075	0.142 *
Vulnerability—COVID	0.447 ***	0.473 ***	0.248 ***	0.035	0.113	0.033
Vulnerability—War	0.156 *	0.235 ***	0.192 **	0.084	−0.022	0.090
Mortality—COVID	0.283 ***	0.383 ***	0.175 **	0.123	0.168 *	0.035
Mortality—War	0.225 ***	0.349 ***	0.168 *	0.059	0.090	0.084
CBI—COVID	0.331 ***	0.464 ***	0.254 **	0.040	0.223 ***	0.123
CBI—War	0.140 *	0.246 ***	0.120	−0.022	0.092	0.059
ISLES—COVID	0.447 ***	0.509 ***	0.248 ***	0.142 *	0.147 *	0.040
ISLES—War	0.339 ***	0.366 **	0.199 **	0.053	−0.035	−0.022
ERQ—Cognitive Reappraisal	−0.032	0.013	−0.145 *	0.068	0.139 *	0.142 *
ERQ—Expressive Suppression	0.315 ***	0.196 **	−0.027	0.077	0.080	0.053

Notes: CBI = Core Belief Inventory; ISLES = Integration of Stressful Life Experiences Scale; ERQ = Emotion Regulation Questionnaire; GP-CORE = General Population–Clinical Outcomes in Routine Evaluation; PHQ-4 = Four-item Patient Health Questionnaire; BSMAS = Bergen Social Media Addiction Scale. * *p* < 0.05, ** *p* < 0.01, *** *p* < 0.001.

**Table 7:** Partial correlation coefficients between mental health variables and relational variables, controlled for satisfaction for life (SWLS).

	GP-CORE	PHQ-4	BSMAS	Risk Behaviors Pre-Pandemic	Risk Behaviors during Pandemic	Risk Behaviors Last Month
RQ—Secure Attachment	0.017	−0.072	0.163	0.042	0.053	0.077
RQ—Fearful Attachment	0.271 ***	0.286 ***	0.201 **	0.083	0.058	0.120
RQ—Preoccupied Attachment	0.217 ***	0.230 ***	0.100	0.066	0.005	0.077
RQ—Dismissive Attachment	0.014	−0.085	−0.056	0.098	0.001	0.032
RQ—Attachment Anxiety	0.298 ***	0.315 ***	0.101	0.008	0.007	0.047
RQ—Attachment Avoidance	0.099	0.011	−0.049	0.035	0.003	0.002
NRI-BSV—Mother Support	0.049	0.115	0.096	−0.141 *	−0.064	−0.030
NRI-BSV—Neg. Int. w Mom	0.024	0.235 ***	0.253 ***	0.144 *	0.174 *	0.145 *
NRI-BSV—Father Support	0.044	0.008	0.140	−0.065	−0.009	−0.002
NRI-BSV—Neg. Int. w Dad	−0.048	0.324 ***	0.300 ***	0.185 **	0.230 ***	0.245 ***
NRI-BSV—SameSexFriendSupport	0.144 *	0.202 **	0.304 ***	0.118	0.153 *	0.165*
NRI-BSV—Neg.Int.SameSeFriend	−0.100	0.099	0.139 *	0.008	0.081	0.033
NRI-BSV—Op. Sex Friend Support	0.221 ***	−0.025	0.111	0.038	0.110	0.121
NRI-BSV—Neg.Int.wOp.SexFriend	0.049	0.087	0.111	0.020	0.068	0.097
NRI-BSV—Partner Support	0.041	0.026	0.129	0.123	0.192 **	0.190 **
NRI-BSV—Neg. Int. w Partner	−0.007	0.071	0.182 **	0.125	0.270 ***	0.257 ***
NRI-BSV—Adult Support	−0.063	0.129	−0.047	−0.067	−0.036	−0.062
NRI-BSV—Neg. Int. w Adult	−0.058	0.138 *	−0.030	0.009	0.011	0.107
Relation w teachers pre-pandemic	0.030	−0.066	−0.040	−0.053	−0.155	−0.046
Relation w teachers during COVID	0.005	−0.089	−0.024	−0.031	−0.110	−0.086
Relation w teachers last month	0.009	−0.067	0.048	−0.012	0.011	−0.020

Notes: GP-CORE = General Population–Clinical Outcomes in Routine Evaluation; PHQ-4 = Four-item Patient Health Questionnaire; BSMAS = Bergen Social Media Addiction Scale. * *p* < 0.05, ** *p* < 0.01, *** *p* < 0.001.

**Table 8:** Significant predictors of mental health indices, weighted for satisfaction for life (SWLS).

Outcomes	Predictors	β	t	Sig.
GP-CORE R^2^ = 0.566	Vulnerability—COVID	0.353	5.799	<0.001
	ISLES—COVID	0.297	5.366	<0.001
	ERQ—Expressive Suppression	0.169	3.399	<0.001
	COVID-19 positive test	0.157	3.336	0.001
	NRI-BSV—Neg Interaction with Father	0.147	3.044	0.003
	RQ—Attachment Avoidance	0.128	2.716	0.007
	NRI-BSV—Father Support	−0.184	−3.837	<0.001
	NRI-BSV—Partner Support	−0.167	−2.795	0.006
PHQ-4 R^2^ = 0.435	Vulnerability—COVID	0.333	4.771	<0.001
	NRI-BSV—Neg Interaction with Father	0.242	4.546	<0.001
	ISLES—War	0.201	3.523	<0.001
	COVID-19 positive test	0.141	2.702	0.007
	CBI—COVID	0.137	2.008	0.046
	Months of remote schooling	0.116	2.204	0.029
Risk behaviors during pandemic R^2^ = 0.133	NRI-BSV—Neg Interaction with Partner	0.229	3.516	<0.001
	NRI-BSV—Neg Interaction with Father	0.180	2.780	0.006
	CBI—COVID	0.173	2.677	0.008
Risk behaviors last month R^2^ = 0.148	NRI-BSV—Neg Interaction with Father	0.227	3.449	<0.001
	NRI-BSV—Neg Interaction with Partner	0.214	3.257	0.001
			

Notes: CBI = Core Belief Inventory; ISLES = Integration of Stressful Life Experiences Scale; NRI-BSV = The Network of Relationships Inventory: Behavioral Systems Version; RQ = Relationship Questionnaire; GP-CORE = General Population–Clinical Outcomes in Routine Evaluation; PHQ-4 = Four-item Patient Health Questionnaire.

## Data Availability

Data are available on requests from corresponding author.
